# Analysis of Feature Intervisibility and Cumulative Visibility Using GIS, Bayesian and Spatial Statistics: A Study from the Mandara Mountains, Northern Cameroon

**DOI:** 10.1371/journal.pone.0112191

**Published:** 2014-11-10

**Authors:** David K. Wright, Scott MacEachern, Jaeyong Lee

**Affiliations:** 1 Department of Archaeology and Art History, Seoul National University, Seoul, Republic of Korea; 2 Department of Anthropology, Bowdoin College, Brunswick, Maine, United States of America; 3 Department of Statistics, Seoul National University, Seoul, Republic of Korea; University of New England, Australia

## Abstract

The locations of *diy-geδ-bay* (DGB) sites in the Mandara Mountains, northern Cameroon are hypothesized to occur as a function of their ability to see and be seen from points on the surrounding landscape. A series of geostatistical, two-way and Bayesian logistic regression analyses were performed to test two hypotheses related to the intervisibility of the sites to one another and their visual prominence on the landscape. We determine that the intervisibility of the sites to one another is highly statistically significant when compared to 10 stratified-random permutations of DGB sites. Bayesian logistic regression additionally demonstrates that the visibility of the sites to points on the surrounding landscape is statistically significant. The location of sites appears to have also been selected on the basis of lower slope than random permutations of sites. Using statistical measures, many of which are not commonly employed in archaeological research, to evaluate aspects of visibility on the landscape, we conclude that the placement of DGB sites improved their conspicuousness for enhanced ritual, social cooperation and/or competition purposes.

## Introduction

How and why people settle onto landscapes have been focal research questions in archaeology since the advent of the discipline (e.g., [1]–[3]). Post-processual archaeology challenged the assumption that the motivations guiding human behavior can be analytically reduced to testable sets of assumptions [4]. However, it is now widely accepted that a middle ground exists between generating absolute laws of governing human behavior and human agents behaving independent of systems or repeatable principles [5]–[7]. The use of scientific mapping tools can be used to pattern human settlements, the construction of archaeological features and provide insight into how human agency expresses itself onto a landscape.

Monumental architecture is commonly seen as a means for solidifying political hegemony [8]–[10] or promoting social cohesion [11]–[13] within or between communities. In Africa, the explanations for the construction of walled settlements and monumental architecture are as diverse as the continent itself [14]–[17]. The diversity of human populations on the African continent gave rise to different forms of political complexity that exhibit unique characteristics worthy of situating in the global discourse of social ascendancy [18].

**Figure 1:**
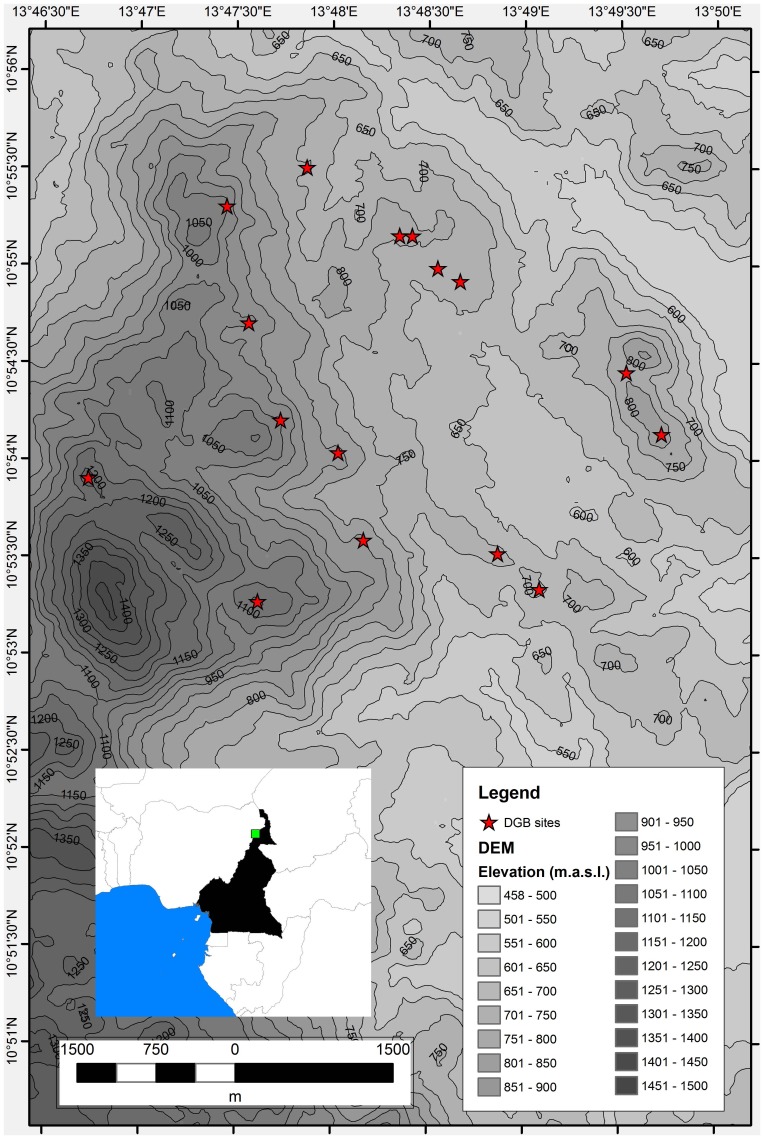
Location of the project area and DGB sites.

In the mid-1990s, a series of tell mound sites at the edge of the Mandara Mountains were excavated in order to study the evolution of social and political systems over the last three millennia (Fig. 1) [19]. The culmination of these cultural developments resulted in the rise of predatory, slave-raiding plains states. More recent research at a set of monumental stone sites (the *diy-geδ-bay* or DGB sites) in the northwestern Mandara massif indicates a parallel history of variable political complexity in the mountains over the last 800 years at least, with some evidence for occupation at least a century or two before that [20]. Archaeological and historical data strongly indicate that the relationship between mountains and plains was extremely complex, and that both regions offered resources and challenges that mutually influenced political and cultural developments in the other [20]–[22].

The motivations behind the construction of DGB sites remain a source of speculation, with one theory being that they were initially constructed as a response to profound droughts in the 15^th^ and 16^th^ centuries CE [19]. This seems to have been a particularly intense period of occupation at the two largest sites, DGB-1 and DGB-2, but excavations on the former has established that initial stages of construction two place at least two centuries before the period of drought [19]. Based on examination of site locations on 1∶50,000 topographic maps, Nicolas David [23] has hypothesized that the sites were situated non-randomly on high ground in order to maximize their visibility over surrounding terrain and their intervisibility, possibly in the course of ritual and/or political competition between local communities and their leaders. However, this hypothesis of non-random location in the service of intervisibility has heretofore remained untested using modern computing methods.

In this paper, we describe novel ways of determining relative intervisibility of archaeological sites as well as the cumulative viewshed of the surrounding landscape based on a Geographical Information Systems (GIS) analysis. Using DGB sites as our case study, we statistically analyze whether the locations of sites were randomly located above an elevational threshold of 700 m.a.s.l. or were selected in order to maximize their viewshed. The results of our statistical tests reject the null hypothesis that DGB site locations were randomly selected and we argue that although other causative site selection factors cannot be ruled out, the viewscape of the DGB sites was certainly a factor in where sites were constructed.

### Background to the research area: DGB sites

The landscape of the northern Mandara Mountains in northern Cameroon and Nigeria is one of the most densely populated in Central Africa, with densities of up to 220 people/km^2^ in a dry Sudanian environment. It is thus by necessity an almost entirely domesticated landscape, with all of the arable land under agriculture and with virtually every tree and shrub exploited for human needs. The region is extremely diverse ethnically, linguistically and culturally, with more than 20 different languages spoken in an area of about 2000 km^2^, and with a complex sociopolitical history to match.

The 6.35-×-6.5 km area covered in this study includes 16 DGB sites that have been previously identified by archaeologists [20], [23]. These sites are scattered across volcanic inselbergs of the Mandara Mountains, which comprise the northeastern segment of the Cameroon Volcanic Line [24]. The Mandara Mountains are mapped as trachyte and rhyolitic plugs [25]. The topography is highly irregular with steep peaks and dissected valleys. The native vegetation is comprised of mosaics of *Pennistum* sp. grasslands and *Acacia* sp. woodlands and allies [26], but has been heavily anthropogenically modified into an agricultural landscape during the Late Holocene with intensive human settlement [20].

**Figure 2:**
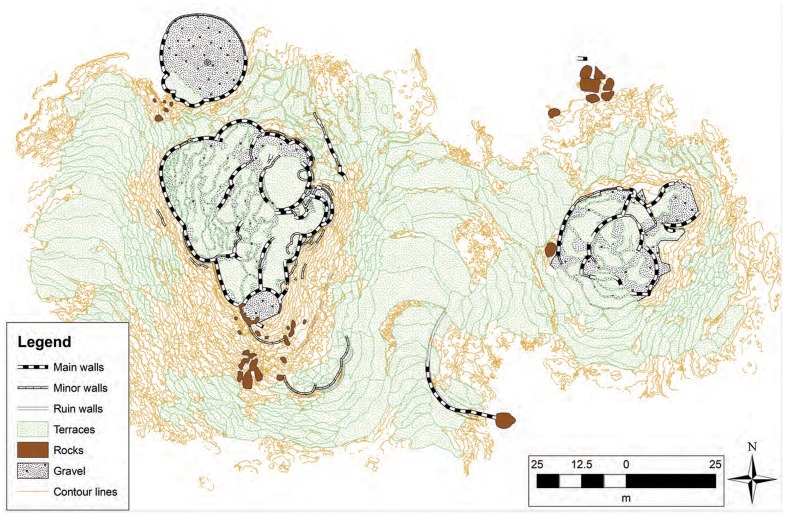
Laser phase scans using a Leica HDS of the DGB-1 and DGB-2 complexes (complements of Heinz Rüther and the Zamani Project).

The 16 DGB sites are prehistoric monumental architectural complexes made up of varying combinations of rubble-filled platforms and terraces, and faced with distinctive and carefully arranged dry-stone facades. A number of them, and all so far excavated, contain a variety of interior passages, staircases, courtyards and chambers. The two largest, DGB-1 and DGB-2, are situated just over 100 m apart and are at least in part contemporary; they thus form a single monumental site complex over an area of about 2.5 ha (Fig. 2.). The other DGB sites are significantly smaller. Seventeen dates from DGB-1 establish occupation during the 13^th^ to 17^th^ centuries AD, while the one reliable date from DGB-2 is from the 15^th^ century [19]. Two dates from DGB-1, the only other excavated DGB site, are of the late 14^th^–15^th^ century AD [23]. It is quite possible that further excavations would increase the period of site usage at these latter sites.

The functions of the DGB sites are not completely understood. They do not appear to have defensive functions, given their positioning, unfortified wall features and lack of close access to water. There is some evidence for contemporary domestic occupation on DGB-1 and in the area between DGB-1 and DGB-2, but they do not seem to have been primarily habitation sites. They provide a striking impression of being in some sense platforms for display, almost for theater: their raised open platforms would certainly afford easy visibility for public activities, while sunken courtyards, subterranean passages and chambers would accommodate private ceremonialism, with stairways linking the two worlds.

David [23] has located the functioning of the DGB sites in the context of modern Mandara montagnard ritual practice, in ceremonialism involving water and fecundity, while MacEachern [27] emphasizes their potential role in the increasingly complex political world of the southern Lake Chad Basin in the mid-second millennium AD, when predatory states and slave-raiding were becoming important elements in the plains north of the Mandara Mountains. The contemporary capital of one of these states, Wandala, was located only 30 km north of the DGB sites, at Keroua/Kirawa on the modern Cameroon-Nigeria border. It is very likely that the DGB sites fulfilled both of these roles simultaneously. Their size and imposing architectural features, along with the labor that would have been invested in their construction, makes it almost certain that they would have played an important role in sociopolitical relationships in the region during their period of use. Given that fact, it will be extremely useful to know if they were situated to maximize their viewsheds and their intervisibility. This would imply that the 16 sites were in fact of an interacting regional cultural system, and might increase the likelihood that some element of comparison, cooperation and/or competition was involved in their use. There are precedents for such regional ceremonialism in the area [28], particularly with the *maray* ceremony held at varying intervals among mountain communities. The ‘passing’ of the ritual between communities might be relevant to questions of intervisibility among ceremonial sites.

The locations of the sites have been previously hypothesized to be situated according to principles of intervisibility [23], potentially for reasons of interregional cooperation and/or competition. Herein, we articulate two sets of testable hypotheses:

(1a) H1: The locations of DGB sites are situated to maximize intervisibility of the sites to one another.

(1b) H0(null): The locations of DGB sites are randomly situated above 700 m.a.s.l. and are not situated to maximize intervisibility to one another.

(2a) H2: The locations of DGB sites are situated to maximize the cumulative viewshed of the surrounding valleys.

(2b) H0: The locations of DGB sites are randomly situated above 700 m.a.s.l. and are not situated to maximize the cumulative viewshed of the surrounding valleys.

By comparing the locations of actual DGB sites (hereafter, Actual DGB) to permutations of stratified random “DGB sites” (hereafter, Random DGB-1, 2, 3…), we intend to accept or reject the hypotheses based on statistical measures of randomness. We are not attempting to eliminate other potential factors for site selection (geology, aspect, soil fertility of adjacent land, visibility for astronomical purposes, etc.), merely testing an earlier hypothesis using the best fit of data generated with modern computing techniques.

### Visibility studies in archaeology

Landscape visibility and feature intervisibility are related concepts in GIS data processing. The landscape visibility is concerned with determining whether features are located in order to see positions at lower elevations, and potentially vice-versa [29]. Methods analyzing feature intervisibility typically determine whether features at relatively similar elevations might have been constructed in their given locations in order to facilitate their ability to see one another. In both cases, the “viewshed” is considered a tangible cultural asset, worthy of enhancing or restricting for some socially important reason. While determining whether or not sites were constructed in order to enhance visibility has become relatively simple, getting at the emic cultural dimensions of feature construction has become no less simple than in the days preceding automated spatial computational techniques.

The intervisibility of archaeological sites or features has been a topic of discussion since the advent of GIS. An early pioneer, Kvamme [30], advocates testing samples of sites against a “background standard” in order to identify random vs. non-random patterns of site placement. Wheatley [31] analyzes the placement Neolithic funerary monuments using a Kolmorgov-Smirnoff (K–S) test of cumulative frequency distributions to argue that the patterns show intentional intervisibility of “long barrow” sites in Stonehenge, but the degree to which the sites are visible to the landscape in general is variable according to region (see also [32] for a similar study on rural settlement in Greece). K–S tests are used to determine whether samples exceed the statistical probability of being randomly distributed. In Kvamme’s [30] analysis, “the sampling frame of…cell values…serves as the background referent distribution”. However, Fisher [33] argues that such studies are most effective if there is a control set of randomized points to compare in order to test random vs. non-random effects. Additionally, in recent years, K–S has been determined to be a less effective test of normality in datasets compared to other non-parametric tests such as Shapiro-Wilk, Lilliefors and Anderson-Darling [34].

A second type of visibility analysis includes the ability of observers to see points on a landscape from archaeological features [33], [35], [36]. This has been called “cumulative viewshed” analysis and has been employed in numerous archaeological studies [30]–[33], [37], [38]. Good visibility of a landscape to and from an archaeological site or feature has been argued to have military applications of site defensibility [39]–[42], resource acquisition [43]–[45] or to solidify some aspect of social power/cohesion through a feature’s visibility [46]–[50]. Regardless of the purported reasons for doing so, it is clear that prehistoric people frequently made informed choices about how to situate archaeological sites to either enhance or reduce visibility relative to points on a landscape.

Early pioneering work on landscape visibility was very labor intensive and lacked statistical rigor because of a lack of data processing tools (e.g., [37], [51]), but Geographic Information Systems (GIS) now provide ample computing power and statistical tools for determining visible and invisible aspects of a landscape from an observer point. The use of Monte-Carlo simulations to create randomized sets of data has become increasingly popular to create control samples of data populations [33], [52]–[54]. The advantage of such simulations is that they remove any possible subjectivity in the analysis, while the disadvantage is that they are difficult to accommodate using stratified random sampling methods.

The fundamental principle of visibility and intervisibility in GIS is to generate lines of sight between given points on a landscape. In the case of two features (Point A, Point B), a line of sight is created as a straight vector between A and B and the topography which would prohibit or facilitate intervisibility is generated from a Digital Elevation Model (DEM) of the terrain. Today, DEMs are commonly available to resolutions of up to 10 m, but most portions of Africa (the subject of this study) are covered by 30-×-30 m grids by United States Geological Survey (USGS) satellites (e.g., http://glovis.usgs.gov). Whether or not Point A is visible to Point B is determined on the basis of analyzing the presence/absence of topographic features that might interfere with the vector connecting the two nodes. Although visibility itself is hindered by physical distance relating to the curvature of the earth and recognizability of objects to the human eye over long distances [35], we presume that the DGB sites were theoretically intervisible on the basis that no two intervisible sites were more than 5 km from each other (which is roughly the distance at which the earth curves out of sight to the naked eye on a continually flat surface).

## Methods

### Spatial organization of the data using GIS

Previous studies of intervisibility have advocated one-sample tests comparing a site sample against a hypothetical “background” sample (the strongest advocate and most commonly cited of these studies is [30]). However, in applying the K–S test, background samples have typically been truly randomized to check distributions of actual vs. hypothetical data to delineate statistical outliers. In the present analysis of DGB sites, we apply a stratified random sampling method for the distribution of hypothetical DGB sites (>700 m.a.s.l.), because a truly randomized distribution of hypothetical sites across the project area would not reflect the true conditions of site selection.

After a DEM of the project area was generated, a spider diagram of DGB sites was created in ArcGIS10.1 as potential sight lines between the facilities. In order to test whether the distribution of DGB sites was situated for intervisibility, random points were generated in a stratified fashion within a polygon shapefile created from a contour above 700 m.a.s.l. This corresponds to the approximate minimum elevation on which DGB sites were located. Therefore, we make the assumption that for any myriad of reasons (e.g., defense, drainage, visibility), there was a cultural proclivity to select locations above 700 m.a.s.l. Ten sets of 16 random points were generated using the “Create Random Points” tool in ArcGIS10.1 with the constraining feature being the elevationally-derived contour line shapefile. A minimum distance of 121 m was assigned between the random points based on the distance between DGB-1 and DGB-2. Lines of sight vectors were constructed using the”v.net.visibility” tool in GRASS6.4. The result was the construction of 120 vectors, which is the total number of lines that interconnect all 16 points (points do not connect to themselves and connections are not made twice). Intervisibility lines were created using the 3D Analyst toolbox (“Line of Sight” tool) from the lines of sight vectors. The DEM was the input surface, and line features were the sight lines created.

Slope of Actual and Random DGB sites were calculated from the raster DEM, then aggregated and averaged to control whether site construction was a function of factors other than intervisibility. Slope values were generated automatically in ArcGIS10.1 by extracting the elevation of plotted DGB points from the Mandara Mountain DEM compared to the eight surrounding raster cells then calculating a % of elevation change. The data were tabulated to show the variance between the slope of Actual and Random DGB sites.

A separate raster map of the observability of DGB sites was created to assess whether site locations were situated so that they were more visible from different landscape points. Based on the resolution of the DEM, the raster cell value was set to include a 30-×-30 m area. Observer points were set as the Actual DGB sites or Random DGB sites according to the point features created randomly. A viewshed analysis was executed according to a simple yes/no (binary) function to determine whether a raster cell has visibility of at least one observer point factoring in potential topographic barriers. The “Viewshed” tool in ArcGIS10.1 determines the number of observer points visible from a given raster cell on the landscape. The values are then classified from high to low with “high” values being a computer-determined value of numerous sites visible from an observer point, and “low” values are those with no visibility to an observer point. The data are then be displayed graphically or exported into table format for statistical analysis.

### Cumulative viewshed analysis

Once stratified random sample sets were created and exported into tabular format, statistical means for testing the distribution of actual vs. random sites were generated to test the hypotheses. Weighted means of cumulative viewsheds [33] were generated from the number of DGB sites (Actual DGB, Random DGB) visible from 30-×-30 m cells within the project area (n = 44084). Spearman’s-ρ correlations were performed on comparing individual DGB sites (Actual and Random) to the computed average of the stratified Random DGB (1–10) sites using R statistical software. Spearman’s-ρ is a measure of concordance between two related, but independent sets of variables, taking into account the related degree of monotonicity of the datasets [55]. Spearman’s-ρ values vary between +1 and −1 depending on the degree of correlation of ranked data. A perfect positive correlation of variables is a +1, a perfect negative correlation is a −1, and a non-correlation value is 0.

### Two-way statistical tests

Additionally, binary sets of intervisible DGB sites were created from lines of sight generated from both Actual DGB and Random DGB points. A Cochran’s Q test was performed on the intervisible points of the 16 DGB sites compared to the 10 permutations of stratified random DGB sites located above 700 m.a.s.l. Cochran’s Q is designed to analyze statistical significance of differences in binary populations [56]. For the purpose of the present study, intervisible sites were classified as “1” and non-intervisible sites were classified as “0”. For example, DGB sites 3 and 4 were intervisible, so they were assigned a 1, while DGB sites 3 and 14 were not intervisible, so they were assigned a 0. McNemar’s contingency tests were performed comparing the randomness of Actual DGB vs. Random DGB-1, 2, 3… as well as Random DGB sites to one another. The McNemar’s contingency tests determine whether two sets of binary data are normally distributed [57], [58]. Such methods have not been applied to spatial data in archaeology, but are common in medical (e.g., [59]) and ecology-related (e.g., [60]) fields.

### Bayesian logistic regression

**Figure 3:**
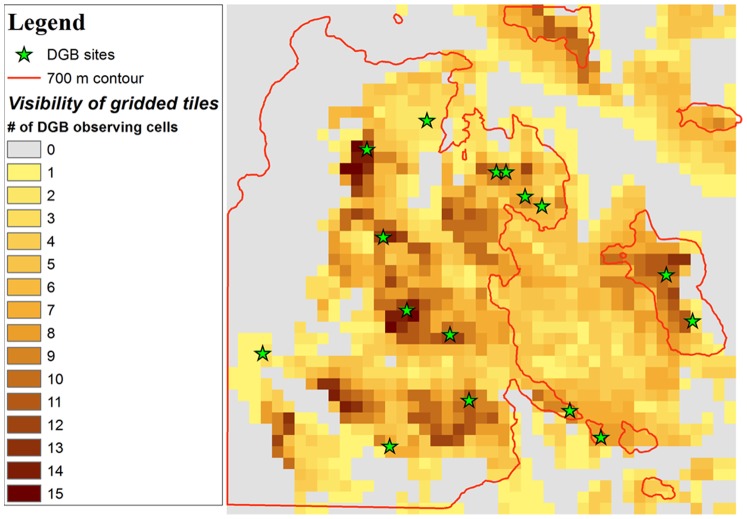
Observation point analysis of 2025 tiles showing the number of Actual DGB sites able to see each cell.

We postulate a Bayesian logistic regression model tests the hypotheses H_0(null)_ and H_1_, i.e. whether the locations of DGB sites are situated to maximize intervisibility of the sites to one another. The test model follows several steps beginning with a spatial organization of the data and concluding with a probability-based calculation. First, we retained the same rectangular-shaped region as previous analyses containing 16 DGB sites and subdivided this region by segmenting the west-to-east line and the south-to-north line into 45 equal length intervals, respectively, resulting in 2025 tiles (Fig. 3). The decision was made to analyze ∼2000 tiles in order to capture enough geographical resolution to have meaningful insight into apparent visibility or non-visibility of sites, but not too much data that would make the analysis overly complex for the 16 DGB sites. The area captured within each tile approximated nine DEM cells plus perimeter values of an additional 16 cells extracted by a rectangular mask in ArcGIS10.1 to estimate the elevation of each 20,375 m^2^ tile.

Among the 2025 tiles within the overall project area, we take n = 1236 tiles located ≥700 m.a.s.l. for the logistical regression analysis. For tile i = 1,2,…,1236, we created two variables, y_i_ and x_i_. We let y_i_ = 1 if a DGB site was built in tile i and y_i_ = 0 otherwise, and let x_i_ be the number of DGB sites that are visible from tile i excluding the DGB site on tile i. If the DGB sites are situated to maximize intervisibility of the sites to one another, the chance of y_i_ = 1 would increase, as x_i_ gets larger.

Second, we postulate the logistic regression model assuming that i = 1,2,…, n, y_i_ follows Bernoulli distribution with probability p(x_i_) which is a function of the number of DGB sites intervisible from tile i. The function p(x) is the probability that a DGB site is built in a tile when there are x DGB sites visible from the tile. The functional form that relates to the probability and the number of visible DGB sites is the logit function; in particular, logit(p(x)) = log [p(x)/(1-p(x))] = α+β x. The parameter α is logit (p) when x = 0, i.e., there are no visible DGB sites from the tile. The parameter β is amount of increase in probability that a DGB site is built in the logit scale as x increases. If β = 0, the number of visible DGB sites does not affect the probability of building DGB site; if β >0, the probability of building DGB sites gets larger as x gets larger; if β <0, the probability of building DGB sites gets smaller as x gets larger.

Since we are interested in whether the number of intervisible DGB sites affect the probability of building DGB sites, we formulate the question in the following statistical hypothesis testing problem:



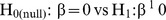



For the prior of α and β, we take α ∼ N (0, 10000) whose variance is very large representing vague prior knowledge on α, and β∼0.5 δ_0_+0.5 N (0, 10000), where δ_0_ is the probability distribution which has probability 1 at 0. The latter prior represents that β is 0 with probability 0.5 and follows N (0, 10000) with probability 0.5. We are effectively putting equal prior probabilities to H_0_ and H_1_, respectively. In the actual implementation of the prior of β, we introduce another random variable γ, which follows Bernoulli distribution with probability 0.5. In particular, γ = 1 if β≠0 and γ = 0 if β = 0. The samples from the posterior distribution of α, β, and γ are obtained by Markov chain Monte Carlo; in particular we use the R2jags package of R statistical language to generate the posterior sample from the posterior.

### Ripley’s K function

Ripley’s K function (multidistance spatial cluster analysis) was run on the projected data points to determine the degree of clustering or dispersion at different distances. The Ripley’s K analysis determines whether the number of neighbors for a set of points at a given distance is greater or less than that of a hypothetical distribution of points [61]. A total of 50 distance bands were generated with a computed confidence envelope of nine permutations per analytical distance. The outer boundary was simulated using the ArcGIS10.1 “Outer Boundary Simulator,” which simulates points outside the study area in order to minimize edge effects. The beginning distance band was 50 m, increasing by 100-m increments in each iteration of the analysis. We applied the “Simulate Outer Boundary Values” using an automatically-generated, minimum-enclosing rectangle for the project area as the feature class of the study area.

The data are not perfectly suited for Ripley’s K analysis because the randomized site locations were stratified with the user-defined minimum elevation of site locations being ≥700 m.a.s.l. However, because the distribution of land ≥700 m.a.s.l. is relatively well distributed across the study area, Ripley’s K patterns were deemed as potentially instructive in showing a site selection bias in the distribution of Actual DGB sites when compared to their stratified random counterparts [53], [54].

## Results

### Ripley’s K

**Figure 4:**
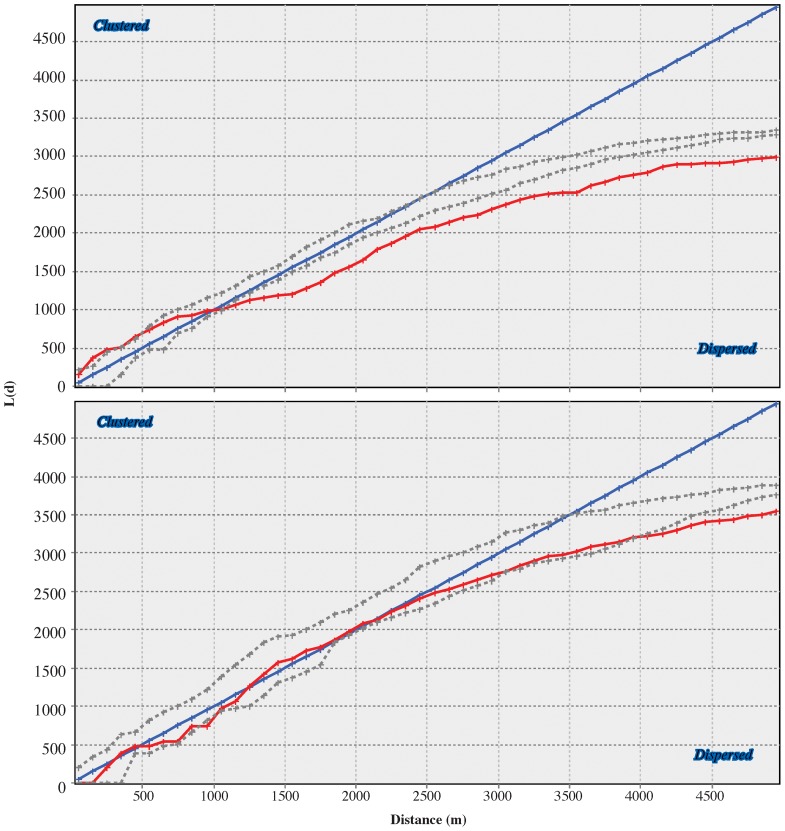
Ripley’s K analysis of clustering of Actual DGB sites (top) and Random DGB-1 (bottom). The blue line represents the predicted values, the red line represents the actual values analyzed and the dotted lines represent the 2-σ confidence envelope.

The various measures of spatial analysis demonstrate that the placement of DGB sites on the landscape for intervisibility concerns was not random. Ripley’s K analysis can be highly sensitive to the size of the project area being evaluated [62], [63], and the results of our analysis bear this out. The Ripley’s K analysis demonstrates clustering of Actual DGB sites at the scale of ≤300 m (exceeding the 95% confidence envelope), a moderate degree of clustering ≤1000 m (within the 95% confidence envelope), but dispersal at >1000 m (Fig. 4). In contrast, the randomly placed points simulating the potential DGB sites (Random DGB 1 was selected for the figure, but all results are similarly distributed) show a random distribution ≤2500 m with dispersion thereafter (Fig. 4). Dispersion measured >2500 m is statistically significant, but analytically insignificant as the project area itself is only 39.7 km^2^. Therefore, dispersion measured above the 2500-m scale in Random DGB sites reflects the effects of the boundaries in the simulation, as the computations include hypothetical neighbors outside the project area itself. When compared to the 10 permutations of stratified Random DGB sites, the clustering of the Actual DGB sites ≤300 m and moderate clustering between 300 and 1000 m is provisionally interpreted as a function of a necessary principle of intervisibility-the sites need to be within view of each other and dispersion of the sites would hamper observers’ abilities to see other sites. The clustered pattern of Actual DGB sites ≤1000-m scale is interpreted to reflect the apparent preference to locate DGB sites in locations in which other DGB sites are visible. However, the 10 permutations of Random DGB sites do not reflect this bias, so, predictably, their distribution is not patterned to accommodate this concern.

### Cochran’s Q and McNemar’s contingency tests

**Figure 5:**
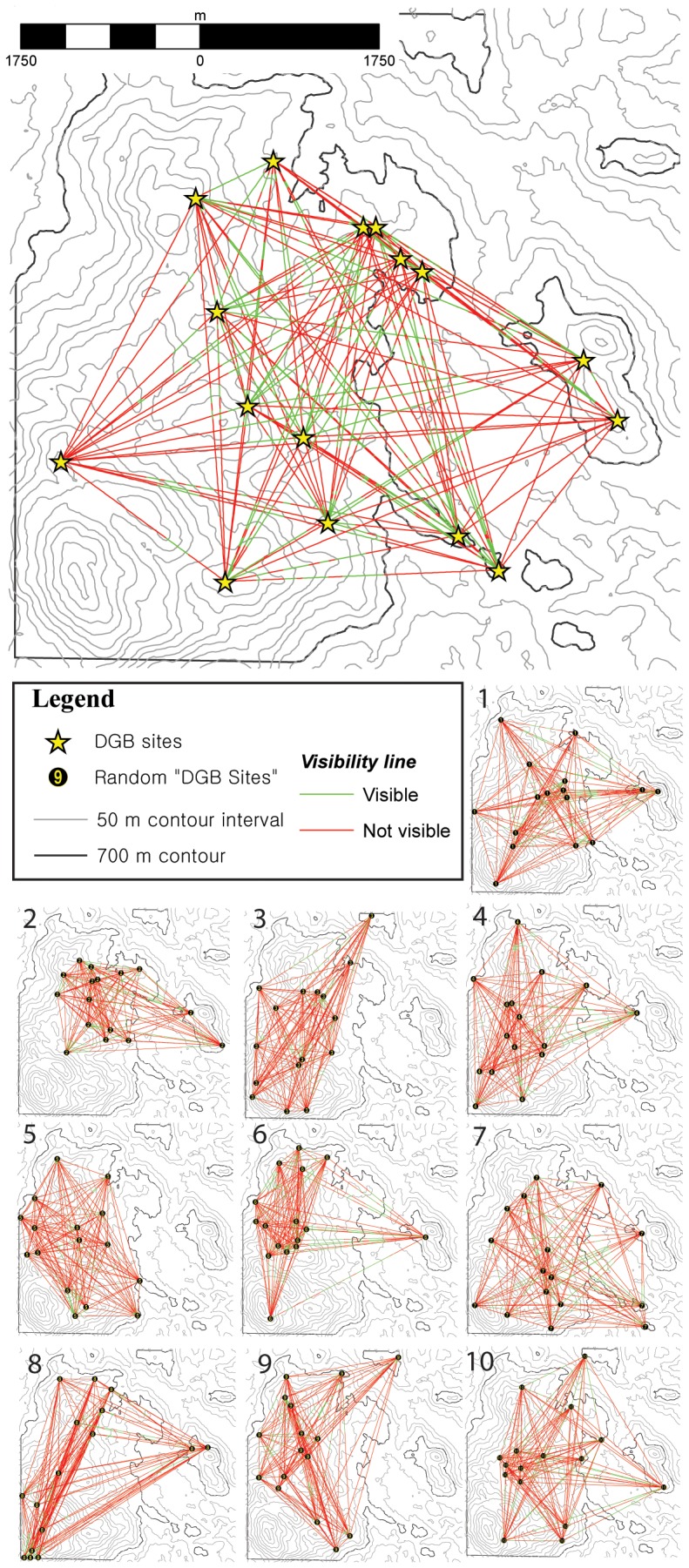
Intervisibility lines generated using the “Line of Sight” tool in ArcGIS10.1 Spatial Analyst (vectors created using v.net.visibility in GRASS6.4) of Actual DGB and 10 permutations of Random DGB sites.

In order to further test this assumption, a strict measure of the degree to which the placement of sites was related to their ability to see one another was generated. The comparison of the intervisibility of Actual DGB sites compared to the 10 permutations of random sites was performed using the Cochran’s Q test (Fig. 5; Table S1). The test statistics of the 11 sites (n = 240) yielded a Cochran’s Q value of 444.502 (df = 10, asymptotic significance = 0.000). With only two degrees of freedom (comparing Actual DGB sites with Random DGB-1 and Random DGB-2), the Cochran’s Q value was 98.709 (asymp. sig. = 0.000). When removing the Actual DGB sites from the Cochran’s Q, the value was 59.621 (df = 9, asymp. sig. = 0.000); while with two degrees of freedom (comparing Random DGB-1, −2 and −3), the value was 15.955 (asymp. sig. = 0.000). We interpret the asymptotic significance of Cochran’s Q among the Random DGB sites as reflecting the bimodal nature of the intervisibility of the stratified random sample with Random DGB-1, −2, −6, −7 and −8 having 10 or more intervisible sites and Random DGB-3, −4, −5, −9 and −10 having six or less intervisible sites. The stratified random sampling of the test DGB sites skewed intervisibility of sites higher than a truly random distribution, but not as high as the actual distribution of DGB sites.

Therefore, in order to test whether there was a systematic bias in selecting stratified random DGB sites, a McNemar contingency test was performed comparing all stratified random samples (Random DGB-1– Random DGB-10) dichotomously with Actual DGB sites. The results are shown in Table S2 and demonstrate that the differences between the actual distribution of intervisible DGB sites and stratified random samples are statistically significant above the 99% confidence envelope. Furthermore, five permutations of McNemar’s test were performed on the samples. The samples selected were non-biased (1 vs. 2, 3 vs. 4, 5 vs. 6, 7 vs. 8, 9 vs. 10) and demonstrate no statistical difference in the intervisibility of sites at the 95% confidence interval (Table S2). The results clearly demonstrate that the stratified random selection of hypothetical DGB sites did not inherently bias the intervisibility of sites against the actual distribution of sites.

### Spearman’s-ρ test

**Figure 6:**
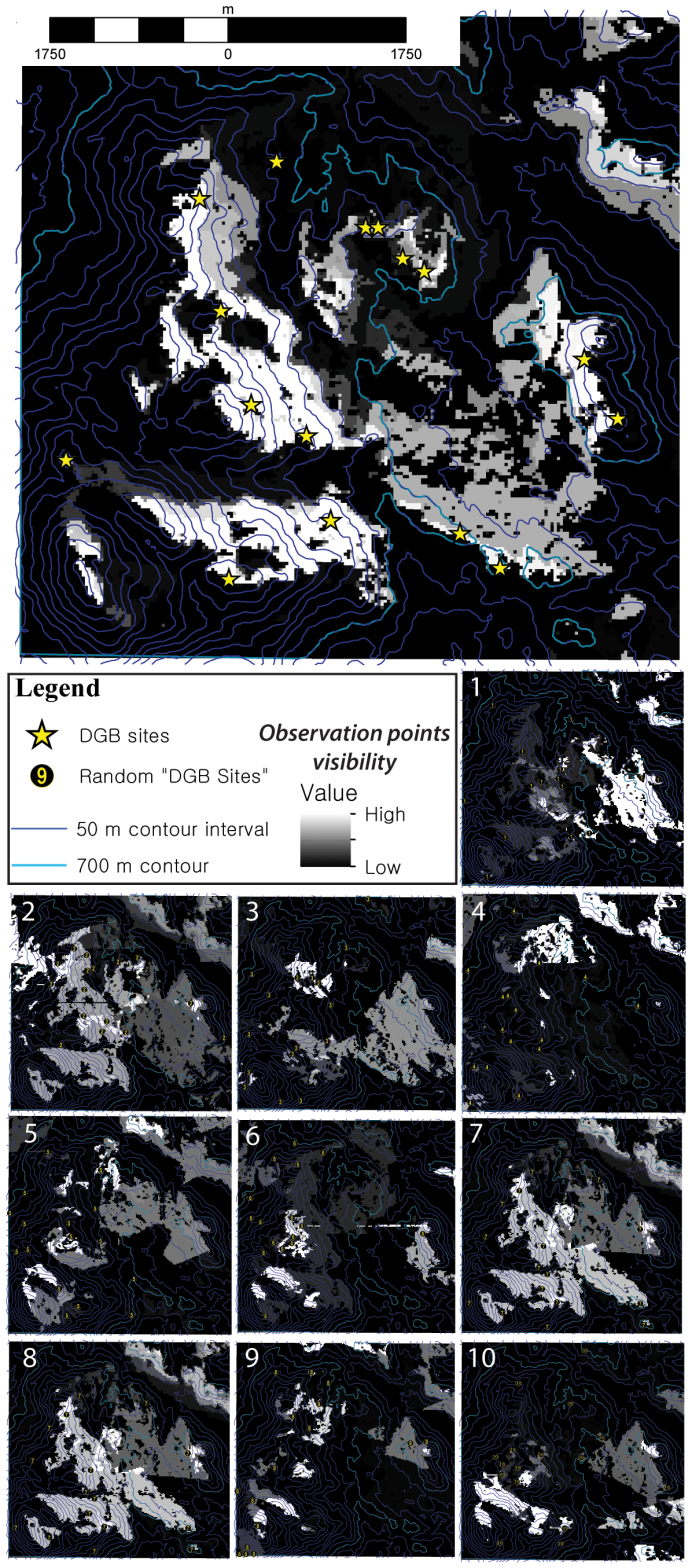
Viewshed analysis in ArcGIS 10.1 of Actual DGB and 10 permutations of Random DGB sites.

**Figure 7:**
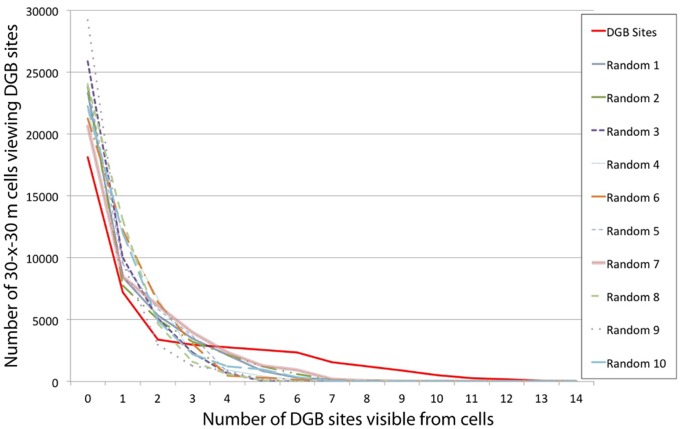
Comparison of the number of DGB sites (Actual, Random-1– Random-10) visible from 30-×-30-m grid cells within the study area.

Analyzing landscape visibility (a.k.a. cumulative viewshed) of Actual DGB sites vs. Random DGB-1– Random DGB-10, shows that Actual DGB sites command a higher visibility of the surrounding landscape compared to the test sites (Fig. 6; Fig. 7; Table 1). The Spearman’s-ρ analysis shows almost no differences in the ranks of data between Actual and Random DGB sites and an average of the stratified random DGB sites. A Spearman’s-ρ analysis of ranks of the number of sites visible from individual 30-×-30 m grid cells from the Actual and Random DGB sites vs. the average ranks of Random DGB-1– Random DGB-10 (Table 2) show strong positive relationships between the ranks of data. All R values were >0.9 with Random DGB-9 having the weakest correlation coefficient and Random 1, 2, 7 and 10 showing perfect (+1) positive correlations. Actual DGB sites had R-values of 0.98 and τ-values of 18.74, which wasn’t significantly different than the average values. We interpret the strong positive relationships between these data as reflecting the relatively good aspect of visibility of sites >700 m.a.s.l. selected in the stratified random sampling method undertaken in this study.

**Table 1:** Comparative analysis of numbers of DGB sites (Actual, Random-1-10) visible from 30-×-30-m grid cells within the study area.

Number ofSites Visible	DGBActual	Random-1	Random-2	Random-3	Random-4	Random-5	Random-6	Random-7	Random-8	Random-9	Random-10	mean(ALL)	1-σ	mean(Random-1-10)
0	18132	23372	23846	25894	21873	24090	21274	20721	23996	29189	22240	23147.9	2884.3	23649.5
1	7226	8427	7782	10028	11853	9532	12254	8468	13043	9609	12017	10021.7	1991.1	10301.3
2	3384	5329	5029	5111	6716	5863	6434	6136	4678	2985	4945	5146.4	1170.5	5322.6
3	2961	3474	3218	2363	2135	3691	3069	3985	1612	1253	2209	2724.5	874.6	2700.9
4	2762	2145	2178	635	917	780	488	2359	605	865	1240	1361.3	830.5	1221.2
5	2545	845	1215	47	434	102	312	1272	117	183	991	733.0	755.2	551.8
6	2362	313	606	6	150	24	126	916	30	0	340	443.0	698.6	251.1
7	1568	132	163	0	6	2	87	172	3	0	79	201.1	458.4	64.4
8	1227	38	44	0	0	0	40	46	0	0	21	128.7	364.8	18.9
9	906	9	3	0	0	0	0	9	0	0	2	84.5	272.5	2.3
10	499	0	0	0	0	0	0	0	0	0	0	45.4	150.5	0
11	250	0	0	0	0	0	0	0	0	0	0	22.7	75.4	0
12	185	0	0	0	0	0	0	0	0	0	0	16.8	55.8	0
13	60	0	0	0	0	0	0	0	0	0	0	5.5	18.1	0
14	17	0	0	0	0	0	0	0	0	0	0	1.5	5.1	0
MEDIAN	1568	132	163	0	6	2	87	172	3	0	79	201.1	458.4	64.4
1-σ	4601.7	6173.7	6221.5	6936.5	6211.0	6493.1	6120.0	5561.9	6767.7	7681.3	6218.9	6229.3	807.0	6438.6
invis. km^2^	16.3	21.0	21.5	23.3	19.7	21.7	19.1	18.6	21.6	26.3	20.0	20.8	2.6	21.3
visible km^2^	23.4	18.6	18.2	16.4	20.0	18.0	20.5	21.0	18.1	13.4	19.7	18.8	2.6	18.4
**weighted avg.**	**2.3**	**1.0**	**1.1**	**0.7**	**0.9**	**0.8**	**0.9**	**1.3**	**0.7**	**0.5**	**0.9**	*1.0*	*0.5*	0.9

Invisible vs. visible km^2^ represent the total area that can see ≥1 DGB site. Weighted average multiplies the number of sites visible by the number of cells visible to and from the sites divided by the total number of cells in the project area. Note: Total cells analyzed are 44,084; each cell is 900 m^2^.

**Table 2:** Spearman’s-ρ ranked analysis comparing the average cumulative viewshed of cells from DGB Random-1–10 against individual values.

Average DGB Random 1–10 vs.	Spearman's R	Spearman's t	p-level
Actual DGB	0.9820	18.7350	0.0000
Random1	1.0000	0.0000	1.0000
Random2	1.0000	0.0000	1.0000
Random3	0.9389	9.8330	0.0000
Random4	0.9661	13.4907	0.0000
Random5	0.9661	13.4907	0.0000
Random6	0.9860	21.3307	0.0000
Random7	1.0000	0.0000	1.0000
Random8	0.9661	13.4907	0.0000
Random9	0.9027	7.5631	0.0000
Random10	1.0000	0.0000	1.0000
Average*	**0.9734**	**13.9906**	

*Average of Spearman's t includes only those with positive values.

However, a more categorical approach to the data shows that subtle visibility conditions may have played a bigger role than the scaled data analysis suggests. Visible portions of the landscape from Actual DGB sites (23.4 km^2^) was almost 2-σ above the mean distribution compared to Random DGB-1– Random DGB-10. Additionally, while there were no instances of portions of the landscape where ≥10 Random DGB sites were visible, there were 1011 cells totaling 0.9 km^2^ where ≥10 Actual DGB sites were visible. The weighted average of visible cells on the landscape of Actual DGB sites (2.3 sites per cell) was >2-σ above the next highest permutation of Random DGB-2 (1.1 sites per cell). The results demonstrate that not only were intervisible locations factored into the construction of Actual DGB sites, but the ability of DGB sites to view the surrounding landscape (and probably vice versa) appears to have been a consideration.

### Testing with Bayesian logistic regression model

**Figure 8:**
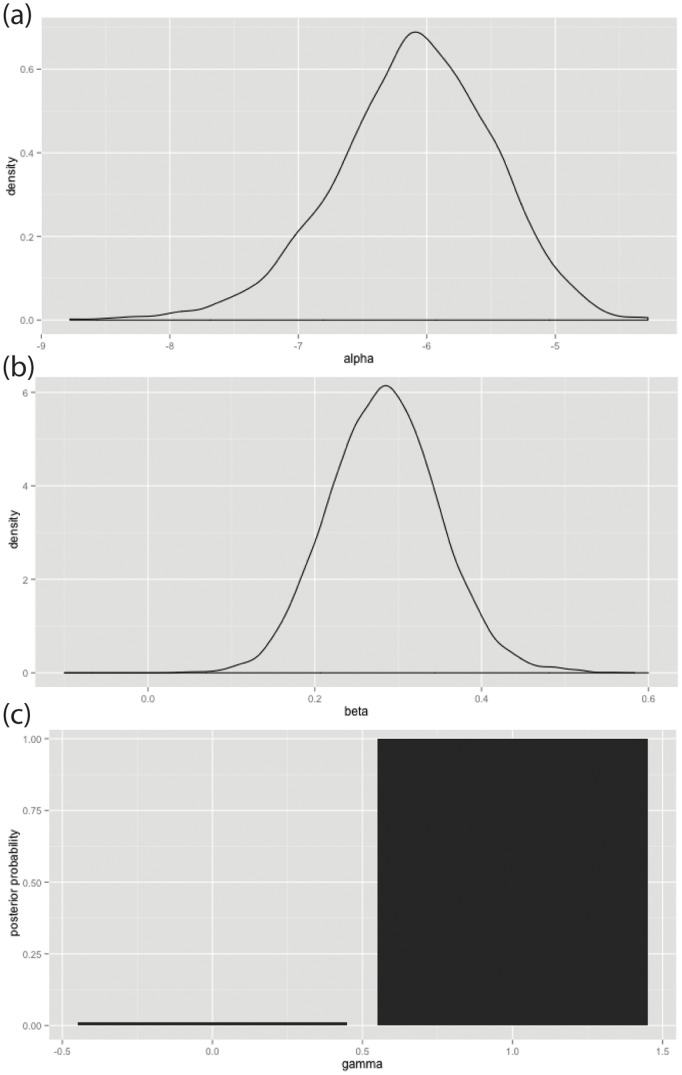
Posterior means and standard deviations of α, β, and γ using a Bayesian logistical regression model to test the intervisibility DGB sites across 2025 tiles, each of which measures 20,375 m^2^.

The posterior means and standard deviations of α, β, and γ calculated from the posterior samples are −6.1014, 0.2828, 0.9990 and 0.61727, 0.07143, 0.03161, respectively (Fig. 8). The posterior probability of β≠0 or equivalently γ = 1 is 0.0001. Since we used the equal prior probability for H_0(null)_ and H_1_, the ratio of the posterior probabilities of H_0(null)_ and H_1_ is the same as the Bayes factor B_10_ and we obtained log B_10_ = 6.90. According to Kass and Raftery’s [64] interpretation of the Bayes factor, this is decisive evidence of H_1_; furthermore, the posterior probability of β >0 is 0.999. On that basis, we conclude that rejecting H_0(null)_ indicates that the probability of building a DGB site is larger for the tiles with a greater number of intervisible DGB sites.

### Secondary factors (elevation and slope)

Secondary factors of site location were also evaluated pertaining to elevation and slope. Actual DGB sites are ≤1.3-σ below the average elevation of all DGB sites (Actual and Random DGB-1– Random DGB-10; Table 3). However, the slope of Actual DGB sites are 2.1-σ less than average DGB sites (Actual and Random DGB-1– Random DGB-10), indicating that there is a much stronger preference for constructing Actual DGB sites on lower slope than lower elevation. Maintaining a constant elevation of DGB sites is not interpreted as having played a significant role in site selection, as Actual DGB sites are within 1-σ of the mean standard deviation of all DGB sites analyzed in this study. Therefore, principles of intervisibility of DGB sites to each other, the visibility of DGB sites to places on the landscape (and vice versa) as well as the secondary factor of low-slope topography (which may have facilitated and enhanced the first two factors) are interpreted as prime factors in determining the locations of Actual DGB sites in Mandara Mountains of northern Cameroon.

**Table 3:** Average and 1-σ calculations of elevation (m.a.s.l.) and slope (%) of Actual and Random DGB sites.

	DGB	DGB	DGB	DGB	DGB	DGB	DGB	DGB	DGB	DGB	DGB	mean		mean
	Actual	Random-1	Random-2	Random-3	Random-4	Random-5	Random-6	Random-7	Random-8	Random-9	Random-10	(ALL)	1-σ	(Random-1-10)
Average elevation	866.4	890.1	846.5	952.5	967.3	913.5	946.2	917.1	971.6	911.1	918.6	918.3	39.9	923.4
1-σ elevation	157.3	193.4	107.8	187.3	191.9	172.4	170.1	163.5	215.4	122.3	169.3	168.2	31.2	169.3
Average slope	10.9	18.8	20.3	19.9	18.6	16.4	15.0	17.3	20.5	16.0	14.6	17.1	2.9	17.7
1-σ slope	9.2	7.7	9.0	8.9	6.7	9.2	6.2	11.4	10.7	9.6	7.6	8.7	1.6	8.7

## Discussion

Based on the GIS and related statistical analyses of the locations of DGB sites, the null hypotheses (H_0_–the locations of DGB sites are randomly located above 700 m.a.s.l.) can be rejected. The statistical evidence demonstrates that there is >99% probability that good intervisibility of DGB sites and/or the surrounding landscape was considered when deciding where to construct sites. The GIS analysis confirms previous research hypotheses [23] that the inferred intervisibility of DGB sites was significant, possibly for maintaining relations of cooperation and/or competition among the communities farming the adjacent hill-slopes. DGB sites retain community ritual and patrimonial functions today [65], and the archaeological evidence suggests that their earliest functions were related at least in part to ritual use [19].

As cited above, intervisibility studies of point features on archaeological landscapes are increasingly common because of their relative ease using GIS computational techniques. We employ relatively novel statistical measures for archaeological research (Cochran’s Q, McNemar’s, Bayesian logistic regression) to crosscheck the effectiveness of our stratified randomization of potential DGB sites within a circumscribed study area. The advantage of two-way tests over K–S tests is that they are designed to compare binary tables rather than means of distribution. Since the analytical design of the intervisibility study was to compare identical distributions of points, means testing are not suitable to test the hypotheses.

In this study, we reject the null hypothesis that the Actual DGB sites are randomly located, but based on the Cochran’s Q analysis, we must also reject the null hypothesis for the Random DGB sites. However, we argue that the virtue of the stratified random sample augmented the inherent intervisibility of the sites in the sample area. For this analysis, we stress that the actual value of the Cochran’s Q test provides the significance of the difference between the Actual and Random DGB sites. In this case, given the relative degrees of freedom analyzed, inclusion of the Actual DGB sites yielded Cochran’s Q values 5–7× higher than when only Random DGB-1– Random DGB-10 were analyzed. McNemar’s contingency tests individually comparing the Actual DGB sites to each of the Random DGB sites confirms that the actual distribution of sites is significantly different than random sites, while the same test performed on Random DGB sites to one another did not show significant differences. These results are further supported with Bayesian logistic regression, which tests whether there is a mathematical preference for site construction within highly visible portions of the landscape.

Weighted means and Spearman’s-ρ analysis of the cumulative viewshed shows that DGB sites were either positioned to see or be seen from the surrounding landscape. Stratified random permutations of DGB sites above 700 m.a.s.l. have significantly smaller cumulative viewsheds than the Actual DGB sites. The Ripley’s K analysis shows clustering of DGB sites ≤1000 m, and the clusters appear to have been oriented to maximize the cumulative as well as intervisible viewshed.

Possible consideration for a high viewshed is the fact that sites are located on low slope benches of the Mandara massif, which also coincidentally commanded better views of the surrounding areas than any of the Random DGB sites. Site slope has been previously determined to be a high predictor of archaeological settlement, because people do not like to construct settlements in high-slope environments [66]–[68]. Although this explanation suffices for explaining cumulative viewsheds, it does not explain the intervisibility of the features to one another.

These analyses do not exclude other selective factors, such as geology, soil fertility, or social boundaries, but they do demonstrate that seeing and/or being seen by other DGB sites and from points on the surrounding landscape featured prominently in the choice of locations for these sites. As already noted, there are a number of modern ceremonial cycles still practiced in the region that encompass multiple communities, and where intervisibility could well have played a part in some aspects of the ritual, especially in determining when the beginning of the ceremony was ‘passed’ from one community to another [28]. While the details of, for example, the modern *maray* ceremonial cycle do not fit with the architectural details of the DGB sites, *maray* at least provides a conceptual framework for understanding interacting, community-based rituals in the area. In addition, it is quite likely that at least some of the sites, and especially the DGB-1/DGB-2 complex, played a sociopolitical role involving conspicuous display – and conspicuousness is, of course, closely related to visibility and intervisibility.

## Conclusions

Cumulative viewshed and two-way analyses of archaeological sites from northern Cameroon compared to 10 permutations of stratified random sites above 700 m.a.s.l. within a restricted study area concludes that placement of the sites was non-randomly situated to enhance intervisibility between sites, and visibility between the sites and the surrounding landscape. A Bayesian logical regression model also rejects the null hypothesis that site construction location was random according to principles of enhanced visibility. Although other contingent settlement factors cannot be excluded, the locations of the sites were ideally located for maximizing the viewshed of DGB sites, and this likely relates to the interpreted social functions of the sites.

Ripley’s K analysis shows a clustering ≤200 m (above the 95% confidence envelope) and moderate to weak degree of clustering 200–1000 m (within the 95% confidence envelope) reflecting the placement of the sites to maximize the view of intervening areas within the Mandara Mountains. Spearman’s-ρ analyses and weighted means of 30-×-30-m grid cells to Actual DGB sites is ∼2-σ above the mean for all 11 permutations of cumulative visibility analyzed. Furthermore, intervisibility of the sites was demonstrated using binary statistical techniques directly comparing the Actual DGB sites vs. permutations of Random DGB sites. Both Cochran’s Q and McNemar contingency tests indicate that Actual DGB sites have significantly better intervisibility than Random DGB sites in all of the test cases, which is further supported using a logistic regression model.

It is not clear how many of the DGB sites were contiguously utilized, although the available radiocarbon dates indicate some degree of contemporaneity. Intervisibility of some of the features does seem to have affected their placement within the Mandara Mountains along with placement of the sites on lower slope portions of the inselbergs compared to Random DGB sites. This information will be important in continuing analysis of the functioning of the DGB sites. Available archaeological data indicate that that functioning was complex, probably involving both ritual and sociopolitical roles, and the sites probably acted within both local and regional systems of interaction and competition. The placement of the sites in locations that increase visibility and intervisibility certainly reinforces our appreciation of their significance in cultural systems in the Mandara Mountains in the mid-second millennium AD.

## Supporting Information

Table S1 **Results of Cochran’s Q tests. (a) Actual DGB and Random DGB-1–10, (b) Actual DGB and Random-1–2, (c) Random DGB-1–10, (d) Random DGB-1–3.** Cochran’s Q tests are binary with the number of non-intervisible sites coded as 0, while the number of intervisible sites are coded as 1.
(DOCX)

Table S2 **McNemar’s test comparing (a) Actual DGB sites vs. 10 independent permutations of DGB Random sites and (b) permutations of Random DGB sites against each other.**
(DOCX)
