# Supplementary material for: Analysis of Feature Intervisibility and Cumulative Visibility Using GIS, Bayesian and Spatial Statistics: A Study from the Mandara Mountains, Northern Cameroon
Source: PLoS One. 2014 Nov 10;9(11):e112191. doi: 10.1371/journal.pone.0112191 (PMC4226507; doi:10.1371/journal.pone.0112191)
Supplement: Table S1 — Results of Cochran’s Q tests. (a) Actual DGB and Random DGB-1–10, (b) Actual DGB and Random-1–2, (c) Random DGB-1–10, (d) Random DGB-1–3. Cochran’s Q tests are binary with the number of non-intervisible sites coded as 0, while the number of intervisible sites are coded as 1. (DOCX) [file pone.0112191.s001.docx]

Table 1. Results of Cochran’s Q tests. (a) Actual DGB and Random DGB 1 – 10, (b) Actual DGB and Random 1 – 2, (c) Random DGB 1 – 10, (d) Random DGB 1 – 3. Cochran’s Q tests are binary with the number of non-intervisible sites coded as 0, while the number of intervisible sites are coded as 1.

| **Frequencies** | | |
| --- | --- | --- |
|  | Value | |
|  | 0 | 1 |
| Actual DGB | 140 | 100 |
| DGB Random 1 | 222 | 18 |
| DGB Random 2 | 216 | 24 |
| DGB Random 3 | 237 | 3 |
| DGB Random 4 | 234 | 6 |
| DGB Random 5 | 237 | 3 |
| DGB Random 6 | 230 | 10 |
| DGB Random 7 | 221 | 19 |
| DGB Random 8 | 222 | 18 |
| DGB Random 9 | 238 | 2 |
| DGB Random 10 | 236 | 4 |
|  |  |  |
| (a) | |  |
| N | 240 |  |
| Cochran's Q | **444.502** |  |
| df | 10 |  |
| Asymp. Sig. | .000 |  |
| (b) | |  |
| N | 240 |  |
| Cochran's Q | **98.709** |  |
| df | 2 |  |
| Asymp. Sig. | .000 |  |
| (c) | |  |
| N | 240 |  |
| Cochran's Q | **59.621** |  |
| df | 9 |  |
| Asymp. Sig. | .000 |  |
| (d) | |  |
| N | 240 |  |
| Cochran's Q | **15.955** |  |
| df | 2 |  |
| Asymp. Sig. | .000 |  |
