# Supplementary material for: Analysis of Feature Intervisibility and Cumulative Visibility Using GIS, Bayesian and Spatial Statistics: A Study from the Mandara Mountains, Northern Cameroon
Source: PLoS One. 2014 Nov 10;9(11):e112191. doi: 10.1371/journal.pone.0112191 (PMC4226507; doi:10.1371/journal.pone.0112191)
Supplement: Table S2 — McNemar’s test comparing (a) Actual DGB sites vs. 10 independent permutations of DGB Random sites and (b) permutations of Random DGB sites against each other. (DOCX) [file pone.0112191.s002.docx]

Table 2. McNemar’s test comparing (a) Actual DGB sites vs. 10 independent permutations of DGB Random sites and (b) permutations of Random DGB sites against each other.

(a)

| **Actual DGB vs. Random 1** | | | | | | | |  | |  | | |  | | |  | | |  | | |  | |  |  |
| --- | --- | --- | --- | --- | --- | --- | --- | --- | --- | --- | --- | --- | --- | --- | --- | --- | --- | --- | --- | --- | --- | --- | --- | --- | --- |
| Actual DGB | | Random 1 | | | | | |  | |  | | |  | | |  | | |  | | |  | |  |  |
|  |  | 0 | | | 1 | | |  | |  | | |  | | |  | | |  | | |  | |  |  |
| 0 | | 128 | | | 12 | | |  | |  | | |  | | |  | | |  | | |  | |  |  |
| 1 | | 94 | | | 6 | | |  | |  | | |  | | |  | | |  | | |  | |  |  |
|  | |  | | |  | | |  | |  | | |  | | |  | | |  | | |  | |  |  |
| **Actual DGB vs. Random 2** | | | | | | | |  | |  | | |  | | |  | | |  | | |  | |  |  |
| Actual DGB | | Random 2 | | | | | |  | |  | | |  | | |  | | |  | | |  | |  |  |
|  |  | 0 | | | 1 | | |  | |  | | |  | | |  | | |  | | |  | |  |  |
| 0 | | 124 | | | 16 | | |  | |  | | |  | | |  | | |  | | |  | |  |  |
| 1 | | 92 | | | 8 | | |  | |  | | |  | | |  | | |  | | |  | |  |  |
|  | |  | | |  | | |  | |  | | |  | | |  | | |  | | |  | |  |  |
| **Actual DGB vs. Random 3** | | | | | | | |  | |  | | |  | | |  | | |  | | |  | |  |  |
| Actual DGB | | Random 3 | | | | | |  | |  | | |  | | |  | | |  | | |  | |  |  |
|  |  | 0 | | | 1 | | |  | |  | | |  | | |  | | |  | | |  | |  |  |
| 0 | | 138 | | | 2 | | |  | |  | | |  | | |  | | |  | | |  | |  |  |
| 1 | | 99 | | | 1 | | |  | |  | | |  | | |  | | |  | | |  | |  |  |
|  | |  | | |  | | |  | |  | | |  | | |  | | |  | | |  | |  |  |
| **Actual DGB vs. Random 4** | | | | | | | |  | |  | | |  | | |  | | |  | | |  | |  |  |
| Actual DGB | | Random 4 | | | | | |  | |  | | |  | | |  | | |  | | |  | |  |  |
|  |  | 0 | | | 1 | | |  | |  | | |  | | |  | | |  | | |  | |  |  |
| 0 | | 136 | | | 4 | | |  | |  | | |  | | |  | | |  | | |  | |  |  |
| 1 | | 98 | | | 2 | | |  | |  | | |  | | |  | | |  | | |  | |  |  |
|  | |  | | |  | | |  | |  | | |  | | |  | | |  | | |  | |  |  |
| **Actual DGB vs. Random 5** | | | | | | | |  | |  | | |  | | |  | | |  | | |  | |  |  |
| Actual DGB | | Random 5 | | | | | |  | |  | | |  | | |  | | |  | | |  | |  |  |
|  |  | 0 | | | 1 | | |  | |  | | |  | | |  | | |  | | |  | |  |  |
| 0 | | 139 | | | 1 | | |  | |  | | |  | | |  | | |  | | |  | |  |  |
| 1 | | 98 | | | 2 | | |  | |  | | |  | | |  | | |  | | |  | |  |  |
|  | |  | | |  | | |  | |  | | |  | | |  | | |  | | |  | |  |  |
| **Actual DGB vs. Random 6** | | | | | | | |  | |  | | |  | | |  | | |  | | |  | |  |  |
| Actual DGB | | Random 6 | | | | | |  | |  | | |  | | |  | | |  | | |  | |  |  |
|  |  | 0 | | | 1 | | |  | |  | | |  | | |  | | |  | | |  | |  |  |
| 0 | | 136 | | | 4 | | |  | |  | | |  | | |  | | |  | | |  | |  |  |
| 1 | | 94 | | | 6 | | |  | |  | | |  | | |  | | |  | | |  | |  |  |
|  | |  | | |  | | |  | |  | | |  | | |  | | |  | | |  | |  |  |
| **Actual DGB vs. Random 7** | | | | | | | |  | |  | | |  | | |  | | |  | | |  | |  |  |
| Actual DGB | | Random 7 | | | | | |  | |  | | |  | | |  | | |  | | |  | |  |  |
|  |  | 0 | | | 1 | | |  | |  | | |  | | |  | | |  | | |  | |  |  |
| 0 | | 131 | | | 9 | | |  | |  | | |  | | |  | | |  | | |  | |  |  |
| 1 | | 90 | | | 10 | | |  | |  | | |  | | |  | | |  | | |  | |  |  |
|  | |  | | |  | | |  | |  | | |  | | |  | | |  | | |  | |  |  |
| **Actual DGB vs. Random 8** | | | | | | | |  | |  | | |  | | |  | | |  | | |  | |  |  |
| Actual DGB | | Random 8 | | | | | |  | |  | | |  | | |  | | |  | | |  | |  |  |
|  |  | 0 | | | 1 | | |  | |  | | |  | | |  | | |  | | |  | |  |  |
| 0 | | 129 | | | 11 | | |  | |  | | |  | | |  | | |  | | |  | |  |  |
| 1 | | 93 | | | 7 | | |  | |  | | |  | | |  | | |  | | |  | |  |  |
|  | |  | | |  | | |  | |  | | |  | | |  | | |  | | |  | |  |  |
| **Actual DGB vs. Random 9** | | | | | | | |  | |  | | |  | | |  | | |  | | |  | |  |  |
| Actual DGB | | Random 9 | | | | | |  | |  | | |  | | |  | | |  | | |  | |  |  |
|  |  | 0 | | | 1 | | |  | |  | | |  | | |  | | |  | | |  | |  |  |
| 0 | | 138 | | | 2 | | |  | |  | | |  | | |  | | |  | | |  | |  |  |
| 1 | | 100 | | | 0 | | |  | |  | | |  | | |  | | |  | | |  | |  |  |
|  | |  | | |  | | |  | |  | | |  | | |  | | |  | | |  | |  |  |
| **Actual DGB vs. Random 10** | | | | | | | |  | |  | | |  | | |  | | |  | | |  | |  |  |
| Actual DGB | | Random 10 | | | | | |  | |  | | |  | | |  | | |  | | |  | |  |  |
|  |  | 0 | | | 1 | | |  | |  | | |  | | |  | | |  | | |  | |  |  |
| 0 | | 138 | | | 2 | | |  | |  | | |  | | |  | | |  | | |  | |  |  |
| 1 | | 98 | | | 2 | | |  | |  | | |  | | |  | | |  | | |  | |  |  |
|  | |  | | |  | | |  | |  | | |  | | |  | | |  | | |  | |  |  |
| Test Statistics | | | | | | | | | | | | | | | | | | | | | | | | | |
|  | Actual DGB vs. Random 1 | | | Actual DGB vs. Random 2 | | Actual DGB vs. Random 3 | | | Actual DGB vs. Random 4 | | | Actual DGB vs. Random 5 | | | Actual DGB vs. Random 6 | | | Actual DGB vs. Random 7 | | Actual DGB vs. Random 8 | | | Actual DGB vs. Random 9 | | Actual DGB vs. Random 10 |
| N | 240 | | | 240 | | 240 | | | 240 | | | 240 | | | 240 | | | 240 | | 240 | | | 240 | | 240 |
| Chi-Square^a^ | 61.896 | | | 52.083 | | 91.248 | | | 84.794 | | | 93.091 | | | 80.827 | | | 64.646 | | 63.087 | | | 92.245 | | 90.250 |
| Asymp. Sig. | .000 | | | .000 | | .000 | | | .000 | | | .000 | | | .000 | | | .000 | | .000 | | | .000 | | .000 |
| (b)   \| **Random 1 vs. Random 2** \| \| \| \| --- \| --- \| --- \| \| Random 1 \| Random 2 \| \| \| 0 \| 1 \| \| 0 \| 199 \| 23 \| \| 1 \| 17 \| 1 \| \|  \|  \|  \| \| **Random 3 vs. Random 4** \| \| \| \| Random 3 \| Random 4 \| \| \| 0 \| 1 \| \| 0 \| 231 \| 6 \| \| 1 \| 3 \| 0 \| \|  \|  \|  \| \| **Random 5 vs. Random 6** \| \| \| \| Random 5 \| Random 6 \| \| \| 0 \| 1 \| \| 0 \| 227 \| 10 \| \| 1 \| 3 \| 0 \| \|  \|  \|  \| \| **Random 7 vs. Random 8** \| \| \| \| Random 7 \| Random 8 \| \| \| 0 \| 1 \| \| 0 \| 207 \| 14 \| \| 1 \| 15 \| 4 \| \|  \|  \|  \| \| **Random 9 vs. Random 10** \| \| \| \| Random 9 \| Random 10 \| \| \| 0 \| 1 \| \| 0 \| 234 \| 4 \| \| 1 \| 2 \| 0 \|   Test Statistics | | | | | | | | | | | | | | | | | | | | |  |  |  |  |  |
|  | | | Random 1 vs. Random 2 | | | | Random 3 vs. Random 4 | | | | Random 5 vs. Random 6 | | | Random 7 vs. Random 8 | | | Random 9 vs. Random 10 | | | |  |  |  |  |  |
| N | | | 240 | | | | 240 | | | | 240 | | | 240 | | | 240 | | | |  |  |  |  |  |
| Chi-Square^a^ | | | .625 | | | |  | | | |  | | | 0.000 | | |  | | | |  |  |  |  |  |
| Asymp. Sig. | | | .429 | | | |  | | | |  | | | 1.000 | | |  | | | |  |  |  |  |  |
| Exact Sig. (2-tailed) | | |  | | | | .508^b^ | | | | .092^b^ | | |  | | | .688^b^ | | | |  |  |  |  |  |
| a. Continuity corrected | | | | | | | | | | | | | | | | | | | | |  |  |  |  |  |
| b. Binomial distribution used | | | | | | | | | | | | | | | | | | | | |  |  |  |  |  |
